# The Epidemiology of Q Fever in England and Wales 2000–2015

**DOI:** 10.3390/vetsci4020028

**Published:** 2017-05-19

**Authors:** Kate D. Halsby, Hilary Kirkbride, Amanda L. Walsh, Ebere Okereke, Timothy Brooks, Matthew Donati, Dilys Morgan

**Affiliations:** 1 Emerging Infections and Zoonoses Section, Public Health England, London NW9 5EQ, UK; amanda.walsh@phe.gov.uk (A.L.W.); dilys.morgan@phe.gov.uk (D.M.); 2 Travel and Migrant Health Section, Public Health England, London NW9 5EQ, UK; hilary.kirkbride@phe.gov.uk; 3 Global Public Health, Public Health England, London SE1 8UG, UK; Ebere.okereke@phe.gov.uk; 4 Rare and Imported Pathogens Laboratory, Public Health England, Wiltshire SP4 0JG, UK; tim.brooks@phe.gov.uk; 5 Bristol Regional Laboratory, Public Health England, Bristol BS2 8EL, UK; matthew.donati@phe.gov.uk

**Keywords:** Q fever, surveillance, epidemiology

## Abstract

Between 2000 and 2015, 904 cases of acute Q fever were reported in England and Wales. The case dataset had a male to female ratio of 2.5:1, and a median age of 45 years. Two outbreaks were recognised during this time period, and the incidence of sporadic cases was highest across the southwest of England, and Wales. There are limitations in the surveillance system for Q fever, including possible geographical differences in reporting and limited epidemiological data collection. The surveillance system needs to be strengthened in order to improve the quality and completeness of the epidemiological dataset. The authors conclude with recommendations on how to achieve this.

## 1. Introduction

Q fever is a zoonotic disease caused by infection with the bacteria *Coxiella burnetii*. Its primary animal reservoirs are cattle, sheep, and goats, and it is shed in the urine, faeces, milk, and birth products of infected ruminants [1]. Humans are usually infected through inhalation of dust or aerosols containing the organism, which may be produced during birth or at slaughter [1]. Human cases have also been associated with drinking unpasteurised milk, tick bites, and contact with parturient cats [1]. The majority of acute cases of Q fever in humans are asymptomatic, however the infection may also present as influenza-like illness or pneumonia [2]. Case numbers show seasonal variation with most cases occurring in the spring and summer [3,4], and the pattern is thought to be linked to lambing, environmental contamination, and the movement of livestock [5,6].

The largest recorded outbreak of Q fever in the UK since 2000 occurred at a meat processing plant in Scotland in 2006, involved 142 cases, and was thought to have been caused by airborne transmission from a sheep lairage [7,8]. A subsequent outbreak in the Netherlands between 2007 and 2010, with over 4000 cases, attracted worldwide attention and raised public awareness of the disease [9]. It has resulted in a large amount of work being done to examine Dutch cases of Q fever, whilst cases in England and Wales are less well described.

Q fever is not notifiable in either humans or animals in England and Wales [10], although since 2010 the organism is laboratory reportable in humans [11]. This paper describes the epidemiology of Q fever in England and Wales for the 16 year period from 2000 to 2015.

## 2. Methods

In England and Wales, serologically confirmed cases of *C. burnetii* infection are reported by the source laboratory to Public Health England (PHE). Databases of routine laboratory reports are supplemented with data from the two Q fever reference laboratories (the Regional PHE Laboratory in Bristol and the Rare and Imported Pathogens Laboratory (RIPL) at PHE Porton). The three datasets are combined and de-duplicated using patient name or soundex (phonetic algorithm for indexing names by sound, as pronounced in English), sex, and date of birth.

For the purposes of surveillance and diagnosis, positive test results are divided into three categories: acute infection, chronic infection, and past exposure. Only acute cases are included in this analysis since diagnosis of chronic infection requires clinical information (in addition to microbiological information) which was not available for this analysis. In acute self-limited Q fever, antibodies to phase II antigens appear first, dominate the humoral immune response and are present at higher titre than anti-phase I antibodies. An initial rise in IgM antibody to phase II antigen is followed by an IgG response to the same antigen. Diagnosis of acute infection requires a four-fold rise in titre or a single high titre of >512 (Regional PHE Laboratory in Bristol) or > 640 (RIPL) by immunofluorescence for both IgM and IgG, or a positive PCR in the absence of high phase 1 antibodies.

## 3. Results

**Figure 1:**
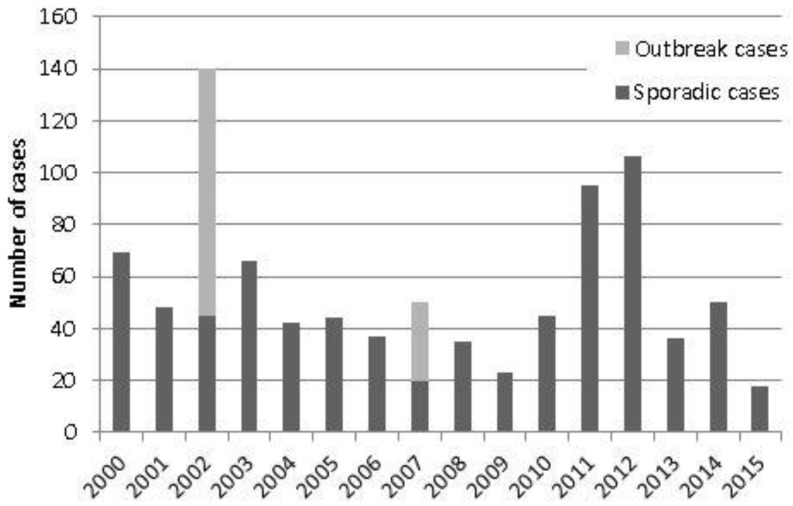
Acute Q fever diagnoses in England and Wales, 2000–2015, by outbreak or sporadic.

A total of 904 cases of acute Q fever were reported in England and Wales between 2000 and 2015 (Figure 1, Table 1). These figures include cases associated with two recognised outbreaks. The first occurred in workers at a cardboard manufacturing plant in South Wales in 2002 (95 cases), where the source was believed to be airborne transmission resulting from drilling into contaminated straw board during office renovation [12]. The second recognised outbreak occurred in Cheltenham in 2007 (30 cases), thought to be due to windborne spread from a farm source [13]. Following the 2002 outbreak, case numbers in England and Wales decreased until 2011 and 2012 when there was a further notable peak. This was mostly due to an increase in cases in the Northwest of England, although a common source was not recognised.

**Figure 2:**
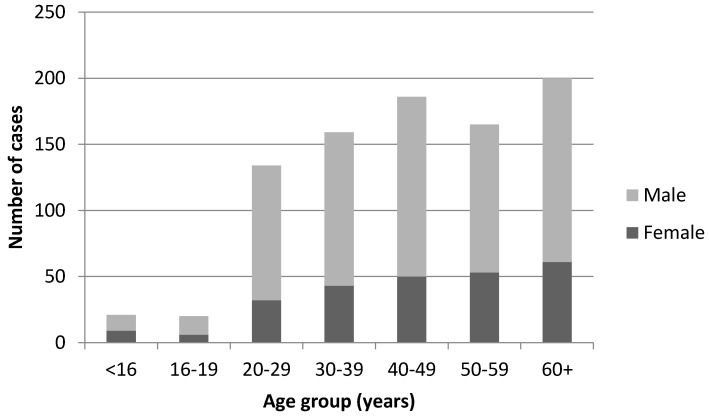
Acute Q fever diagnoses (including outbreak cases) 2000–2015, by age group and sex.

Figure 2 shows diagnoses by age and sex. Six hundred and forty cases (70.8%) were male and 258 (28.5%) were female (ratio 2.5:1). The remaining six cases had unspecified gender. Cases had a median age of 45 years (range 0–100), and 13 cases had unspecified age. Where age was recorded, case numbers for both males and females peaked in the 60+ age group (22.1%). Less than 5% of all cases occurred in children and young adults under 20 years of age (*n* = 42), and 41.1% were over 50 years old (*n* = 366).

In the 2002 outbreak in Wales, 83% of cases were male and there was a median age of 44 years (22–60 years) [12]. Similarly in the 2007 outbreak in Cheltenham, 70% of cases were male with a median age of 48 years (19–72 years) [13].

**Figure 3:**
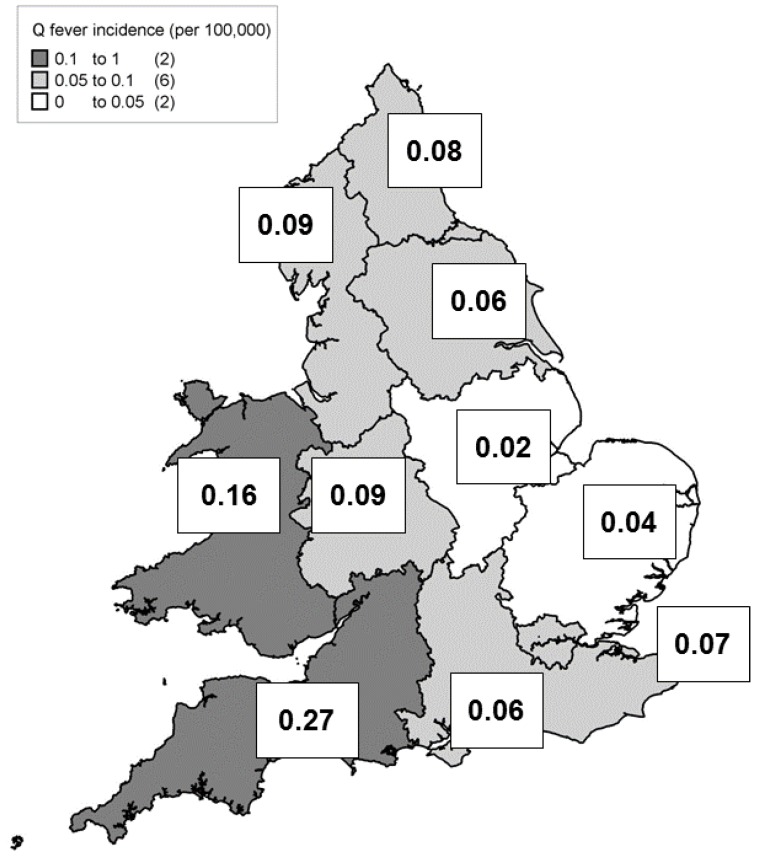
Acute Q fever incidence rates by region in England and Wales, 2000–2015 (excluding outbreak-associated cases). Incidence rates per 100,000 population, using ONS mid-year populations estimates (as at 30 June 2006) [14].

Only 73 cases (8.1%) reported a history of travel between 2000 and 2015, suggesting that the majority of cases reported in England and Wales are autochthonous. The geographical distribution of non-outbreak associated Q fever is shown in Figure 3, with the majority of cases reported from the southwest of England, and Wales. These regions also had the highest mean annual incidences (calculated using ONS mid-year populations estimates (as at 30 June 2006) [14]). The regional distribution of human cases is similar to the distribution and density of sheep and cattle populations [15,16], although the density of human cases is lower in the northern regions of England than the animal populations might suggest.

## 4. Discussion

Between 2000 and 2015, 904 cases of acute fever were reported in England and Wales. This corresponds to a mean annual incidence of 0.09 cases/100,000 population/year (excluding outbreak cases), slightly lower than previously reported figures of 0.1–0.35/100,000 population/year for 1975–1995 [17].

Few cases in this dataset were children (<5% under 20 years) and this supports previous findings that, whilst exposure to *C. burnetii* in childhood is common, children are more likely to have mild or asymptomatic infection and are less likely to be investigated and/or admitted to hospital [18]. As in other studies [19], cases of Q fever in males outnumbered those in females by 2.5:1. This gender difference can be partly explained by the predominance of men in farming and related professions, and there is some support for this from Orr et al.’s 2006 study, which identified a person’s occupation as the most likely route of exposure for sporadic cases [20]. It is also possible that there are underlying biological differences between the genders that can explain the higher rates of infection amongst men. The occurrence of clinical disease in females may be affected by the female hormone 17β-estradiol, which has been found to exert a protective effect for *C. burnetii* infection [21], and a circadian gene (Per2), which is differently modulated in males and females and appears to be related to the clinical status of Q fever patients [22].

There were substantial geographic variations in the distribution of human Q fever within England and Wales. The relatively high rates in the southwest of England may reflect the distribution of sheep in the UK, but may also reflect increased ascertainment due to the location of both reference laboratories. It is not currently clear which source laboratories include Q fever in their serology screens for atypical pneumonia, a policy that could vary geographically. There may be greater awareness of Q fever in the southwest region than elsewhere, and a laboratory survey would aid in understanding laboratory testing practices across England and Wales.

It is also possible that the prevailing south westerly winds in England and Wales may have led to the greater burden of disease in the Southwest and Wales. Unusual changes in wind direction and strength have been implicated as a potential cause of Q fever outbreaks in the past [23].

A retrospective consensus on the case definition for surveillance of acute Q fever in the UK has recently been reached (as detailed in the Methods section) and a definition to include chronic and past cases is being developed for future work (Pers Comms). This is an important step towards standardising diagnostics, and will allow much more reliable interpretation of UK case data. A limitation of this paper was the restriction of the dataset to acute cases only. Diagnosis of chronic infection requires clinical information (in addition to microbiological information) which was not available for this analysis. In addition, chronic and past cases are difficult to assign to a year of onset because they may have acquired their infection several years previously [24]. This restriction to acute cases only will have resulted in an under-representation of the burden of Q fever in England and Wales.

At present, only basic epidemiological information is collected on Q fever patients’ exposure histories, making it difficult to identify the source of infection for many sporadic cases. The authors recommend a period of enhanced surveillance, aimed at more clearly describing the risk factors for Q fever in England and Wales. Work is already underway to describe geographical variation in disease in relation to the distribution of animal populations, and better exposure data is needed to refine this.

An increase in cases was noted in 2011/2012, concentrated in the northwest of England. The reason for this increase is unclear. No human or animal outbreaks were reported, spurious factors such as changes in clinical or laboratory practices were excluded, and no changes in the prevalence of risk factors were identified. The increase is believed to have been either a genuine, unidentified increase in sporadic cases, or an undetected cluster or outbreak. A more robust surveillance system is being developed, to ensure that acute cases are followed up and to enable potential links and sources to be recognised in a timely fashion.

## 5. Conclusions

This paper has described the epidemiology of Q fever in England and Wales between 2000 and 2015. Whilst some case data is available, work is required to improve the quality and completeness of the dataset. The authors recommend:

- Establishing a period of enhanced surveillance, where data on the potential source of an individual’s infection is collected.
- Conducting a laboratory survey to determine the proportion of cases reported to the reference laboratories, and any geographical variation in policy.
- Analysing the geographical variation in the case dataset, to determine whether it is real or ascertainment bias, and to explore reasons for the variation.

In future, it will be possible to compare the data in this paper with the epidemiological data obtained from an improved surveillance system that collects enhanced exposure information. We hope to reflect on the accuracy of this historical data in the light of attempts to improve the quality of the scheme.

## Figures and Tables

**Table 1:** Acute Q fever cases and incidence rates in England and Wales (2000–2015). Incidence rates per 100,000 population, using ONS mid-year populations estimates (as at 30 June 2006) [14].

Year	Total Cases	Sporadic Cases (Excl. Recognised Outbreak Cases)	Incidence Rates (Excl. Recognised Outbreak Cases)
2015	18	18	0.03
2014	50	50	0.09
2013	36	36	0.07
2012	106	106	0.20
2011	95	95	0.18
2010	45	45	0.08
2009	23	23	0.04
2008	35	35	0.07
2007	50	20	0.04
2006	37	37	0.07
2005	44	44	0.08
2004	42	42	0.08
2003	66	66	0.12
2002	140	45	0.08
2001	48	48	0.09
2000	69	69	0.13
Total	904	779	
Mean	56.5	48.7	0.09
